# The Resilience of Hospital Finance

**Published:** 1920-12-11

**Authors:** 


### The Hospital

The Workers' Newspaper of Administrative Medicine and Institutional Life, Administration, National Insurance and Health.

No. 1801, Vol. LXIX. SATURDAY, DECEMBER 11, 1920. PRICE TWOPENCE

### THE RESILIENCE OF HOSPITAL FINANCE.

We must confess that the actual financial position of the London Hospital, as disclosed by the treasurer, Mr. W. T. Paulin, at the quarterly court of Governors, is to us cheering rather than depressing. Struggling against great odds, and faced by expenditure for the current year estimated at £276,329, the governing body have managed to gather together in eleven months of the year the substantial sum of £236,457, and hope to receive before December 31 £8,685 more, the apparent deficit being £31,187. But, as is admitted, no account has been taken of what the December grant of King Edward's Hospital Fund may be (it was £13,000 last year), or of what may come from the distribution of the surplus moneys of the National Relief Funds. It may very well be that the London may, and we hope it will, find itself with a surplus on the year's working. The only disquieting thing, and it is a large factor in the position, is that the hospital started 1920 with a debt of £65,000, which has been financed by pledging free securities. The trust funds apparently have not been hypothecated.

Next year, however, is sure to be critical; the hospital is faced with an increased cost of £10,000 for nursing salaries, but on the other hand there will be new income of at least £20,000 from patients' payments.

We are not too optimistic as to the help from the National Relief Fund. Many a harassed secretary has learned, on reading the conditions of grants, that all that glitters is not gold. Unless a hospital can show a deficit on ordinary expenditure over ordinary income plus free legacies during the five war years it seems that there is little to be hoped for. Moreover, interest on bank loans and deficits of convalescent homes that have had to be met are not to be included in ordinary expenditure. Happy, therefore, is the hospital which has made the tradesmen wait for their money rather than borrow from the bank, and happier still the institution that has not had to carry on its back its convalescent home—part of itself, although not physically connected. Possibly the figures of the London Hospital may be favourable from a National Relief Fund aspect, possibly not—it seems such a matter of chance and accountancy that we do not say too much about it and can only hope for the best.

At the meeting of the British Hospitals Association, fully reported on another page, and called to form a London board of representatives of hospitals to supply a convenient means of approaching and dealing with Government Departments, a letter was read from Charing Cross Hospital stating that that institution is working "on entirely different lines from every other hospital," and begging to be excused taking any part in the proposed movement. It is understood that Charing Cross has weathered the financial tempest very satisfactorily, but, as the letter suggests, it is not altogether income which is the result of the independent attitude, but rather general methods of management. It is, as Col. F. R. Mildmay points out in a letter to *The Times*, rather cruel that other hospitals should not be allowed to associate in this joint effort with this successful institution and to have an opportunity of making themselves acquainted in detail with the nature of methods so beneficent.

The Minister of Health, Col. Mildmay states, has represented to him that the circumstances of the various London hospitals differ so greatly as regards endowments, wealth of the district served, and pecuniary position in general that, of necessity, their requirements must differ considerably. We thoroughly agree with Dr. Addison, and his opinion is greatly strengthened by the foregoing picture of the extraordinary resilient character of the finances of the London Hospital and of the independently prosperous condition of Charing Cross Hospital. These things point to one thing, and that is the wisdom of the appointment of the Committee to inquire into hospital conditions generally. It is most true that the public know very little indeed about these conditions, and a knowledge of the truth will restore a proper sense of proportion and remove the idea, which many potential givers must have, that they would, did they give, only be attempting to salve a fast-sinking vessel.

We hope that the Committee of Inquiry will quickly be appointed, and that the five members chosen will be men of sound judgment who will set to work at once and produce within a very few months an authoritative statement of the hospital position at the present time.

We expect that the solution to the financial problem will be found in an amendment of the National Insurance Act. The voluntary hospital system has too long been the Cinderella of the approved societies, and the time has come when the enormous services to this State system rendered by the hospital are recognised. According to Sir Napier Burnett, the amount spent on the Act during 1919 was £17,330,000, and the receipts were £24,400,000, this being for one year only. The estimate of a surplus in the funds of fifty millions does not sound visionary. The difficulties of the hospitals began, suggests Sir Napier Burnett, at the passing of the Act; insurance patients have flocked for treatment without any hospital benefiting. Millions of those who pay premiums under the Act have received no help from it at all, but to those who receive the service of the Insurance system it is likely, if the Act is amended, that institutional benefit will be as satisfactory a return for the money paid as any of the benefits of the National Health as it now stands.

